# Identification, Classification and Characterization Analysis of *FBXL* Gene in Cotton

**DOI:** 10.3390/genes13122194

**Published:** 2022-11-23

**Authors:** Jingwen Pan, Muhammad Zulfiqar Ahmad, Shouhong Zhu, Wei Chen, Jinbo Yao, Yan Li, Shengtao Fang, Tengyu Li, Akwasi Yeboah, Liangrong He, Yongshan Zhang

**Affiliations:** 1 College of Agronomy, Tarim University, Alar 843300, China; 2 State Key Laboratory of Cotton Biology, Institute of Cotton Research, Chinese Academy of Agricultural Sciences, Anyang 455000, China

**Keywords:** *FBXL* family, cotton, gene expression, phylogenetic

## Abstract

*F-box/LR* (*FBXL*), Leucine-rich repeats in F-box proteins, belongs to the Skp1-Cullin1-F-box protein (SCF) E3 ligase family. *FBXL* genes play important roles in plant growth, such as plant hormones, responses to environmental stress, and floral organ development. Here, a total of 518 *FBXL* genes were identified and analyzed in six plant species. Phylogenetic analysis showed that *AtFBXL*s, *VvFBXL*s, and *GrFBXL*s were clustered into three subfamilies (Ⅰ-Ⅲ). Based on the composition of the F-box domain and carboxyl-terminal amino acid sequence, FBXL proteins were classified into three types (Type-A/-B/-C). Whole-genome duplication (WGD) along with tandem duplications and segmental contributed to the expansion of this gene family. The result indicates that four cotton species are also divided into three subfamilies. *FBXL*s in cotton were classified into three clades by phylogenetic and structural analyses. Furthermore, expression analyses indicated that the expression patterns of *GhFBXL*s in different cotton tissues were different. The highly expressed of *GH_A07G2363* in 5–8 mm anthers, indicates that this gene might play a role in the reproductive process, providing candidate genes for future studies on cotton fertility materials. This study provides an original functional opinion and a useful interpretation of the FBXL protein family in cotton.

## 1. Introduction

The intracellular proteins in eukaryotes are degraded through the ubiquitin/26S proteasome system (UPS) [1]. The substrates of UPS were recruited in the ubiquitin ligase complex which is generated by the interaction of F-box proteins with the following: kinetochore protein 1 suppressor (Skp1), Cullin 1 (CUL1), and Ring-Box 1 (RBX1) [2]. The 40–50 amino acid-based F-box domain is present at the N-terminus of F-box proteins and interacts with Skp1. The F-box proteins’ name originated from the identification of the first N-terminal region of cyclin F [3]. The highly variable protein—protein interaction (PPI) domains such as WD40 repeats, Kelch repeats, and Leucine-rich repeats (LRR) are present at the C-terminus of the F-box proteins. These domains interact with their specific target molecules [4]. Several SCF component-related F-box proteins have been identified [5,6], but the F-box protein also functions with non-SCF complexes [7,8,9]. In eukaryotes such as yeast, flies, nematodes, humans, and plants, many F-box proteins have been identified in the past few years [5,6,10,11,12,13,14]. The subfamily “FBXL” is named for those F-box proteins which contain the LRR domain at the C-terminus of the F-box protein and are associated with the recognition of substrate, function, and disease association [15]. The FBXL subfamily is present in animals as well as in plants. In mammals, FBXL2 controls the phosphatidylinositol-3-kinase (PI3K) signaling cascade by interacting with the β-subunit of the p85 pool in the proteasomal degradation process [16]. The FBXL2 protein also interacts with the forkhead box M1 (FoxM1) transcription factor in gastric cancer [17], with ubiquitinates Aurora B to inhibit tumorigenesis [18]. The over-expression of FBXL7 in mice is associated with Alzheimer’s disease [19]. These results showed that FBXL proteins play an important role in mammals.

In plants, various kinds of FBPs have been characterized which play an important role in regulating different cell functions like nodulation [5,6,13], cell cycle control [20,21,22], photomorphogenesis [23,24], the identity of floral organs [25], self-incompatibility [26,27], leaf senescence [28], circadian rhythms [29,30], and the stress response [31,32]. The wheat FBXL protein “TaFBXL” interacts with the TaGPI-AP protein in the nucleus and plasma membrane for degradation through the 26S proteasome system. It is suggested that the TaGPI-AP protein is regulated by the TaFBXL protein [33]. In *Arabidopsis*, the F-box motif is very important for vein patterning at the cotyledon because an over-expression of FBXL produced the cotyledon with abnormal vein patterns [34]. The LRR domain in FBXL targets the suitable substrate through PPI, which results in the degradation of ubiquitinated proteins [35,36]. The AXR1 and HVE/CAND affect the cotyledon vein patterning through ubiquitination [37,38]. Moreover, FBXL is only expressed in the vascular tissues suggesting that their role may be associated with vascular tissues. The mutation in the vascular-specific genes i.e., CVP1 and CVP2, caused the abnormal venation pattern in cotyledon [37,38]. The over-expression of the FBXL gene also exhibited an abnormal vein pattern at the Arabidopsis cotyledon [34]. It showed that FBXL proteins have a strong association with cotyledon vein patterning vascular tissues. Additionally, the plant-specific domain, FBD motif, is also present at the C-terminus of the FBXL protein. Although the exact function is unknown of the FBD motif in FBPs, FBXL proteins may be involved in the vein patterning of cotyledon as an FBP depends on the F-box, LRRs, and FBD [34]. 

Cotton has a unique position among fiber-producing crops. Previously, Zhang et al. analyzed then characterized and identified 592 F-box protein encoding genes [39]. In this work, we present the detailed study of the F-box proteins’ subfamily FBXL in four cotton species (*Gossypium hirsutum*, *Gh*; *Gossypium barbadense*, *Gb*; *Gossypium arboreum*, *Ga*; and *Gossypium raimondii*, *Gr*) along with *Arabidopsis thaliana (At)*, and *Vitis vinifera (Vv)*. Our results will provide the first overview of the FBXL protein family in six different plant species which will serve as a foundation in the investigation of the FBXL function in plants for future studies.

## 2. Material and Methods

### 2.1. Identification and Physicochemical Analysis of the FBXL Protein Family Members

This study used the same sequence database of genome and identification methods as our previous study [40], including *Gh*, *Gb*, *Ga*, *Gr*, *At*, and *Vv* [41,42,43,44,45]. 

The original inquiry sequences were the 592 published F-box protein sequences from *G. hirsutum* [46]. The *FBXL* genes with the F-box domain at the N-terminus and LRR at the C-terminal were selected as the candidate gene. *FBXL* candidate genes were identified in *G. hirsutum*, and their amino acid sequences were used as inquiry sequences to search the homologous sequences in the protein databases of four cotton species by blastp software (Blast+, Camacho et al., MD, USA) [47].

Furthermore, the ExPASy tool (http://web.expasy.org/, accessed on 26 September 2021) was used to analyze the physicochemical property (i.e., isoelectric point, molecular weight, and length) of *FBXLs* of cotton, which were identified from the currently available genome database. The use of the CELLO v2.5 (Yu et al., Taiwan, China) server predicated the subcellular localization of *FBXL* genes [48].

### 2.2. Analysis of the FBXLs Conserved Motifs and Gene Structure

FBXL conserved motifs were identified using the MEME (Multiple Em for Motif Elimination) website (http://meme-suite.org/, accessed on 1 March 2022, Bailey, et al., Washington, USA), where the *p*-value of each motif was lower than that of 1 × 10^−5^. FBXLs protein domains were predicted by the SMART database (http://smart.embl-heidelberg.de/, accessed on 1 March 2022, v2020, Letunic, et al., Heidelberg, Germany). The motif, exon/intron structure, and conserved domain of *FBXL* genes in cotton were constructed by TBtools (v1.098661, Chen et al., Guangzhou, China) [49,50,51].

The template file was obtained from the AlphaFold DB (AlphaFold Protein Structure Database, https://alphafold.ebi.ac.uk, accessed on 11 May 2022, Varadi, et al., Hinxton, UK) [52], and the SWISS-MODEL (https://swissmodel.expasy.org/, accessed on 12 May 2022, Waterhouse, et al., Basel, Switzerland) [53] was used to generate the three-dimensional protein structure.

### 2.3. Sequences Alignments and Phylogenetic Analysis

The FBXL proteins’ amino acid sequences were aligned using the default parameters of MUSCLE 3.8.31 (Robert C Edgar, CA, USA) [54]. We used MEGA 7.0 (Kumar, et al., Philadelphia, USA) [55] to construct the maximum likelihood (ML) tree, by finding the best model, the bootstrap tests of 1000 replicates, the WAG + G + F model, the γ Distribution option, and the partial deletion option.

### 2.4. FBXLs in Four Cotton Species Chromosome Locations and Collinearity Analysis

The TBtools software and the BLAST (basic Local Alignment Search Tool) were used to locate the *FBXL* genes on the chromosomes of 4 cotton species and all the protein sequences were incorporated into the local database [51]. The whole protein sequence was used as a query to search the above database, and its e-value was 1 × 10^−5^. The results of blastp were analyzed by MCSCAN, and a collinear block covering the whole genome was produced. The collinear pairs belonging to the FBXL family were extracted and a collinear diagram was drawn by the Circos and TBtools software [47,56,57].

### 2.5. FBXL Gene Family Duplicated Gene Pairs Calculation of Selection Pressure

Using the MEGA 7.0 and the TBtools v1.098661 software simple Ka/Ks calculator operates according to the method published in our previous research, and finally estimated the selection pressure of each duplicate *FBXL* gene pair [40,51,54,55].

### 2.6. Analysis of Cis-Elements Promoter Regions of GhFBXLs

The 2000 bp DNA sequences in upstream regions of *GhFBXL*s were obtained from CottonFGD (https://cottonfgd.org/, accessed on 9 September 2021) as promoters [41]. We searched and analyzed the cis-regulatory elements of the *GhFBXL* gene in the promoter region through the PlantCARE website (http://bioinformatics.psb.ugent.be/webtools/plantcare/html/, accessed on 9 September 2021) [58,59].

### 2.7. Analysis of GhFBXL Genes Expression

RNA-Seq data were obtained from the Cotton Omics Database (http://cotton.zju.edu.cn/10.rnasearch.html, accessed on 15 September 2021) to analyze differentially expressed genes under different tissues [43]. RNA-seq data were evaluated by quantitative RT-PCR (qRT-PCR). Total RNA was extracted from roots, stems, leaves, and anthers (bud size < 3 mm, 4–5 mm, 5–8 mm, and >8 mm) of Zhongmiansuo 100 (*Gossypium hirsutum* L., cultivated in Baibi Town, Anyang, Henan Province, China). The developmental stages of different anthers were divided as described by Koltunow [60] and Scot [61]. All the primer sequences are shown in Table S10. The GhActin gene was selected as the internal control gene. The experiment was repeated 3 times, and the relative expression level of the *GhFBXL* genes was calculated by the 2^−∆ct^ method. The experimental reagents and instruments used were referred to by Zhu et al. [40].

### 2.8. FBXL Proteins Gene Interaction Network Analysis

Based on the homologous genes of Arabidopsis thaliana, the multiple sequence search of STRING network analysis was performed by STRING software (https://string-db.org/, accessed on 19 April 2022, Szklarczyk, et al., Zurich, Switzerland) [62]. The confidence parameter was set to 0.4 thresholds, and the interaction of the FBXL protein was analyzed.

## 3. Results

### 3.1. Identification and Physicochemical Properties of FBXL Genes in Cotton

The 121, 119, 57, and 51 *FBXLs* genes after the blast analysis were obtained from the genome of *Gh*, *Gb*, *Ga*, and *Gr*, respectively (Table S1). The F-box and LRR domain were confirmed in the candidate genes through Interproscan 5 (http://www.ebi.ac.uk/interpro/, accessed on 10 July 2021) and SMART (http://smart.embl.de/, accessed on 10 July 2021) tools. We noticed that the tetraploid *Gh* contains almost double the number of *FBXL* genes as its diploid parents *Ga* and *Gr.* The results suggests that mostly *FBXL* genes remain preserved during polyploidization between *Ga* and *Gr.* Detailed information on physical parameters like transcript ID, protein length (aa), location on chromosome, isoelectric point (pI), protein weight (kDs), and predicted subcellular location of all the *FBXL* genes of four Gossypium species are listed in Table S1. The molecular weight of the FBXL proteins varied from 24.53 (GH_A06G0370) to 111.067 (GH_A07G0202) kDs in *Gh*, from 21.61 (GB_A02G0078) to 115.04 (GB_A11G3348) in *Gb* from 24.65 (Ga02G0228) to 103.64 (Ga06G0927) in *Ga*, and from 24.62 (Gorai.003G025500) to 110.7 (Gorai.001G020200) kDs in *Gri* with an average molecular weight of between 57.38~61.84 (Table S1). It can be seen that the genes in the FBXL family have differences in their molecular weight, amino acid number, and isoelectric points, which leads to subtle differences in the functions of genes. The predicted subcellular localization indicates that FBXL proteins are found in almost all organelles. In addition to the nucleus (20.69%) and plasma membrane (22.13%), other *FBXL* genes localize to the mitochondria, chloroplast, cytoplasmic and extracellular organelles (Table S1).

### 3.2. Domain, Conserved Motif, and Gene Structure Analysis

**Figure 1:**
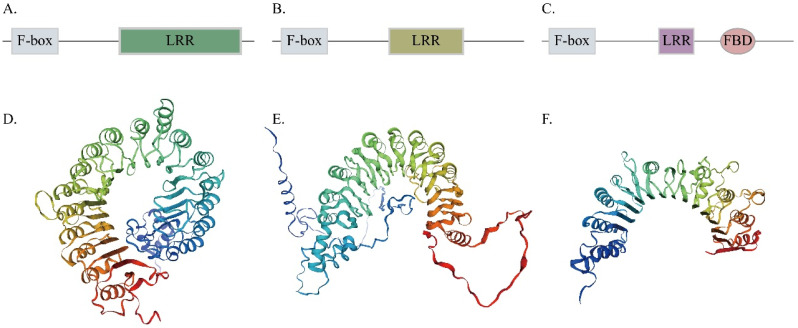
The classification of FBXL proteins. (**A**–**C**) Group-Ⅰ, Group-Ⅱ, and Group-Ⅲ domain structures, respectively. (**D**–**F**) Three-dimensional structure of representative genes of Group-Ⅰ, Group-Ⅱ, and Group-Ⅲ.

Gene function is closely related to the domain arrangement, conserved motifs, and gene structure. The FBXL protein family can be classified into 3 different groups based on their functional domains in the C-terminal region (Figure 1A–C). Groups A and B of the FBXL protein subfamily contain a different number of LRR regions at the C-terminal, and the various LRRs accumulate together to shape the domed docking structure [63]. Group C contains LRR and plant-specific FBD domains. The FBD domain contains about 80 amino acids and its function is supposed to associate with nuclear processes [12]. We selected three Arabidopsis orthologs genes (*TIR1*, *FBL17*, At1g13570) from Groups A, B and C, respectively. The molecular structure file of *Arabidopsis FBXL genes* was downloaded from AlphaFold, and then imported into SWISS-MODEL to build the model (Figure 1D–F). The 3D protein structures show that one α-helix and a β-strand consisted of an LRR [64]. These three proteins are composed of different numbers of LRR; this effectively supports the three types of our classification.

**Figure 2:**
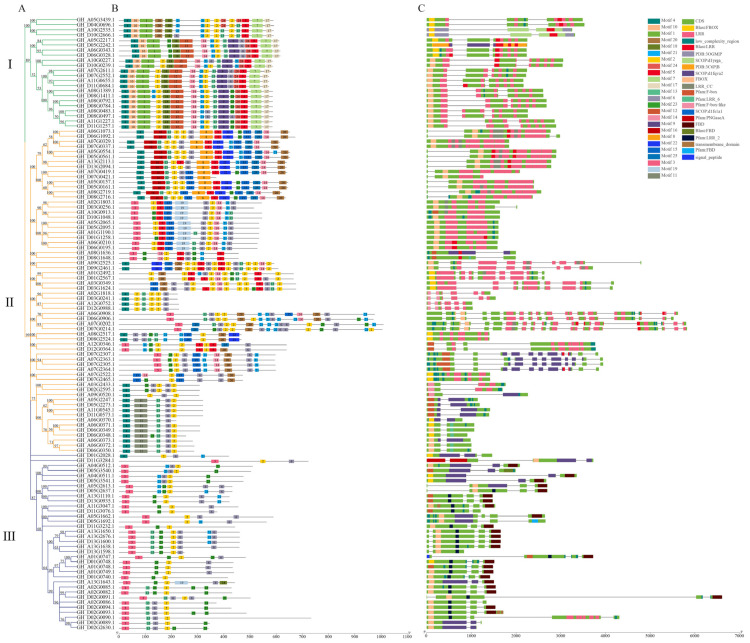
The conserved motifs, exon–intron structures of *GhFBXLs*. (**A**) Phylogenetics tree of *GhFBXL* genes. Used maximum likelihood (ML) method in MEGA7.0 software to create the phylogenetic tree. (**B**) *Gh* FBXL protein Motif prediction. (**C**) Analysis of exon—intron and gene structure of *GhFBXL* genes. (I: Grpup I; II:Group II; III: Group III).

Further, in order to study the existence and conservation of homologous sequences in the two domains, F-box and LRR or LRR/FBD, we used the online MEME tool to make multiple sequence alignments to generate sequence logos of the two domains in the cotton species. Among the 25 motifs identified in *GhFBXL*, 1 motif was found to be conserved in 22 genes of clad-I, 64 genes of clade-II, and 35 genes of clad-III (Figure 2). Motifs 1,7,10 and 17 were only found in clad-I (Figure 2). It is speculated that a certain function has been lost in the evolutionary process. There are consistent structural domains in each category, with semblable structural characteristics. Using the same method to analyze the motifs of *GaFBXL*, and *GrFBXL*, they had similar motifs compared with *GhFBXL*, except for the number of genes that is only about half in *GhFBXL* (Supplementary Figures S1 and S2).

The gene structural analysis was carried out to assess the structural evolution of each *FBXL* gene in three cotton species. The presence of introns in *FBXL* genes ranged from 0 to 18 (Table S2). Based on introns presence, the *FBXL* genes can be split into four classes i.e., intronless, 1-intron, 2-intron, and more than 3-intron at each gene. The maximum *FBXL* genes (34.9%; 80) were observed in the 2-intron class followed by the 3-intron class, which contains 31.9 % genes. The intronless class contains the minimum number (10.5%) of intron regions (Table S2). The distribution of exons and introns of *FBXL* genes are more uniform and consistent within each class. The structural differences among different *FBXL* classes may be due to their unique characteristics and conservation of various components.

### 3.3. Phylogenetic Analysis of FBXL Genes

**Figure 3:**
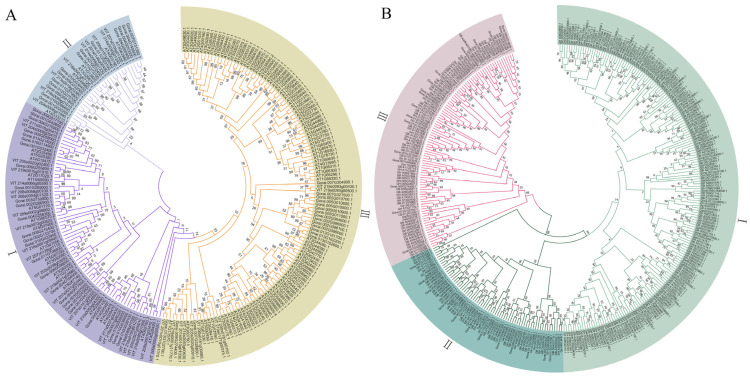
Phylogenetic relationship among FBXL proteins. (**A**) 227 *FBXL* genes from 3 plant species analyzed regarding the phylogenetic relationship. (**B**) 348 *FBXL* genes from 4 cotton species analyzed regarding the phylogenetic relationship. The two maximum likelihood (ML) phylogeny trees were constructed by the MEGA 7.0 software. The bootstrap method was used and replicated 1000 times. (I: Grpup I; II:Group II; III: Group III).

To elucidate the evolutionary relationship of the *FBXL* gene family in *Gossypium* species, the maximum-likelihood approach was applied to construct the phylogenetic tree using 1000 bootstrap replicates with *AtFBXL* (127), *VvFBXL* (43), and *GrFBXL* (57) named as Group-A. The same method was used to remodel the phylogenetic tree from *GhFBXL* (121), *GbFBXL* (119), *GaFBXL* (51) and *GrFBXL* (57), named as Group-B (Figure 3). The phylogenetic tree analysis showed that the *FBXL* members were divided into three monophyletic clades in both trees, in which FBXL family members containing the same or similar C-terminal region fall together in one cluster. In Group-A, the number of *Gh*, *Ga*, *Gr*, and *Gb FBXL* genes in clad-I was almost double that of those in clad-II, and clad-III, the same distribution of *At*, *Vv* and *Gr*, was also observed in Group B (Table S3). The variation in the number of *FBXL* distribution in each clad was found. For example, 71, 29, and 127 *FBXL* members were found in clad-I, clad-II, and clad-III, respectively (Figure 3A). The same pattern of *FBXL* gene distribution was also observed within the cotton species phylogenetic tree (Figure 3B).

### 3.4. Chromosomal Location and Duplication Event of FBXL Genes

To further analyze the characteristics of the *FBXL* family genes, we performed a chromosome location analysis of the four *Gossypium* species of the gene family and drew a physical map of the chromosome distribution of the *FBXL* family genes (Supplementary Figure S2 and Table S7). In the four *Gossypium* species, the *FBXL* family genes were distributed on different and specific chromosomes. The number of genes on chromosomes of *Gh* and *Gb* tended to be the same. The chromosomes D04, D09, scaffold31727_obj_D02, and scaffold467_obj_A13 of *Gh* and chromosomes A09, D04, D09, and scaffold31993_obj of *Gb* contain a minimum of 1 gene. Two to 8 *FBXL* genes in *Gh* and *Gb* range from other chromosomes, respectively (Supplementary Figure S3). Analysis of the *FBXL* genes on different chromosomes of *Ga* revealed that the A genome is consistent with the A genome chromosomes of *Gh* and *Gb*, except for chromosomes A02, A03, A04, A05, A06, A08, A09, and A13. The distribution of the *FBXL* genes on chromosomes has undergone a large number of new additions and losses. Only the number of genes on chromosomes D03, D04, D09, D10, D11, and D12 were the same as that of the *Gh* and *Gb* D genome. There was no correlation between chromosome length and the number of *FBXL* genes in the four cotton species. These results explain the fact that longer chromosomes do not essentially contain a maximum number of *FBXL* genes (Supplementary Figure S3).

**Figure 4:**
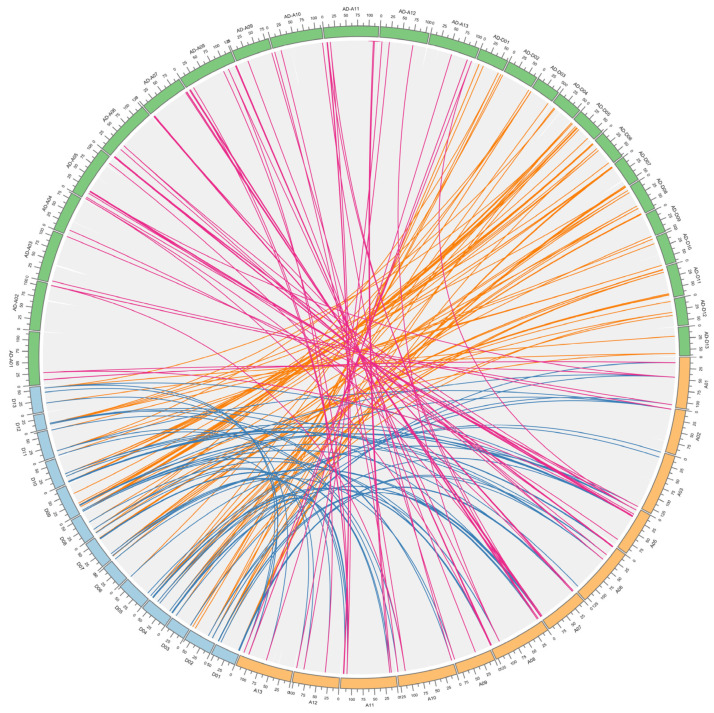
The analysis of synteny and collinearity in *Ga*, *Gr*, and *Gh*. The chromosomes of *Ga*, *Gr*, and *Gh* represents orange, light blue, and grass green colors, respectively. The lines of blue, orange and rose lines represent the GrD/GaA, GhAD/GrD, GhAD/GaA genomes, respectively.

Several cotton genes have been observed to be duplicated at the whole genome level when the A-genome of *Ga* and the D-genome of *Gr* are combined to develop allotetraploid cotton containing the AD genome. The evolution of gene families generally undergoes three processes, namely fragment replication, whole-genome replication, and tandem replication. A joint analysis of the *FBXL* genes of *Gh*, *Gb*, *Gr*, and *Ga* was carried out, and the gene duplication and collinearity between them were analyzed, which indicated that the two tetraploid genomes of *Gh* and *Gb* are composed of two ploidy genomes (*Gr* and *Ga*) during genetic transformation. Homologous sequences can usually be predicted by genomic collinearity analysis, and some functions of homologous sequences might be similar. Therefore, genome-wide collinearity analysis is of great value for function prediction. The genes connected by collinearity lines represent the same gene. Several chromosomes in the GhAt/GhDt, GbAt/GbDt subgenome, and GaA and GrD genomes are connected by lines of the same color, namely, the GhAt/GhDt and GbAt/GbDt subgenomes have *FBXL* homologous genes in the GaA and GrD genomes (Figure 4). These findings indicate that these genomes/subgenomes are closely related during evolution, and most of the *FBXL* genes have been obtained during the evolutionary process of polyploidy. Genes located in the same chromosome region are classified as tandem duplications (TD), while genes from the same genome are known as whole-genome duplication (WGD)/segmental duplications (SD). Observed were 41 *FBXLs*, which arepart of tandem duplicated; 152 *FBXLs*, which are part of WGD/segmental duplicated, and 36 *FBXLs* part of other replication types among 3 cotton species (Tables S4 and S5). The tandem duplicated *GaFBXL* genes are located on Chr01, Chr03, and Chr11, the *GrFBXL* genes on the Chr01, Chr02, Chr05, Chr07, Chr10, and Chr 13, and the *GhFBXL* genes on the A01, A02, A04, A06, A07, A13, D02, D05, and D06. It is suggested that the regularity of the tandem repetition and further verification of the allotetraploid cotton species is derived from the evolutionary hybridization of the diploid cotton species [57]. The number of WGD/SD *FBXL* genes was higher than the TD and SD genes and our findings correlate with previous studies, which were based on the gene duplication events in different *Gh* gene families [46,65].

### 3.5. Ka/Ks Selective Pressure Analysis of FBXL Gene Family

**Figure 5:**
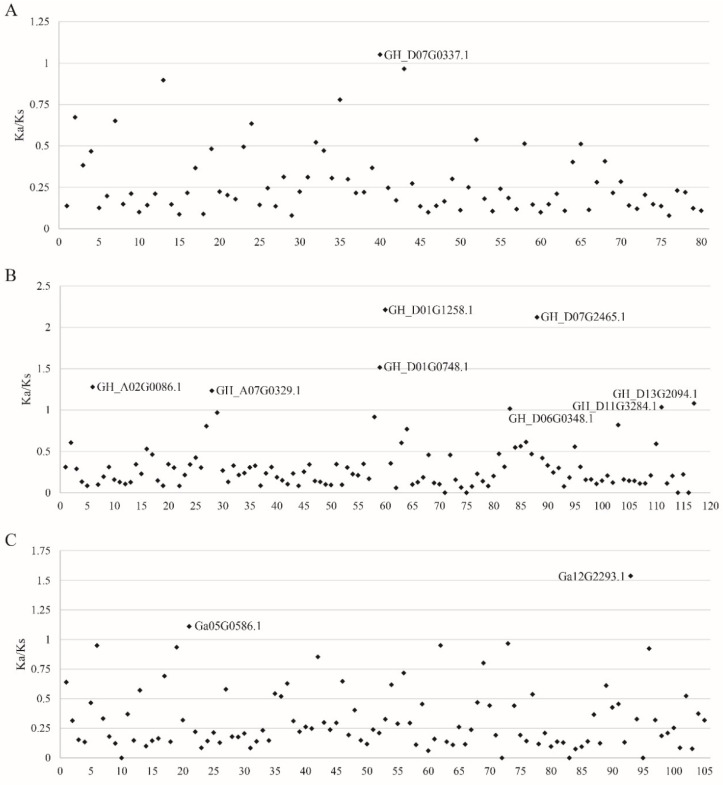
The distribution of Ka/Ks ratio of orthologous pairs between *GhFBXL*, *GrFBXL*, and *GaFBXL*. (**A**) Ka/Ks analysis of *GhFBXL-GhFBXL*. (**B**) Ka/Ks analysis of *GhFBXL-GrFBXL.* (**C**) Ka/Ks analysis of *GhFBXL-GaFBXL*.

The evolution dynamics of the *FBXL* family genes of *G. hirsutum* were explored through comparative analysis of different modes of duplication. This includes the calculation of non-synonymous substitutions/site (Ka), synonymous substitutions/site (Ks), and their ratio (Ka/Ks) of each duplicated pair, resulting in a calculation of the divergence of cotton *FBXL* gene family members. The evolution of *FBXL* family gene pairs of three cotton species was carried out through the selection pressure analysis to observe whether there is selective pressure acting on the FBXL family. In the process of evolution, the duplicated gene pair may also deviate from its original function, which eventually leads to loss of original function (non-functionalization), division of original function (sub-functionalization), and acquisition of new function (new functionalization) [66]. To study whether Darwin’s positive selection is related to *FBXL* gene divergence after duplication, and to determine the nature and degree of selection pressure on these duplication gene pairs, we calculated the Ka and Ks values of duplication gene pairs. These combinations include Gh-Gh, Gh-Gr, Gh-Ga. According to the Ka/Ks ratio, the selection pressure of repeated gene pairs can be inferred. It is generally believed that Ka/Ks = 1 means neutral selection (pseudogene), Ka/Ks < 1 means positive selection effect, and Ka/Ks > 1 means purifying selection effect. A total of 80, 117, and 105 pairs of *FBXL* family genes in the Gh-Gh, Gh-Gr, and Gh-Ga, respectively, were observed. The 1 pair *FBXL* genes in Gh-Gh, 8 pairs in Gh-Gr, and 2 pairs in Gh-Ga were under positive selection effect while others are of purifying selection, indicating that *FBXL* family genes are relatively conservative in the evolutionary process (Figure 5).

Similarly, Gh-Gh, Gh-Gr, and Gh-Ga duplications gene pairs with Ka/Ks values from 0.99 to 0.5 were 10, 13, and 20, respectively (Table S8). The number of duplicated gene pairs with a Ka/Ks ratio from 0.49 to 0 was 69, 96, and 83 (Table S8). Our results suggested that these gene pairs may have undergone rapid evolution after repetition and have experienced positive selection pressure. Since most of the Ka/Ks values were less than 1.0, we predicted that the cotton *FBXL* gene family has experienced strong purification selection pressure and limited functional differentiation after fragment replication and WGD [67] (Figure 5).

### 3.6. Analysis of Cis-Elements in Predicted Promoter Regions of GhFBXLs

**Figure 6:**
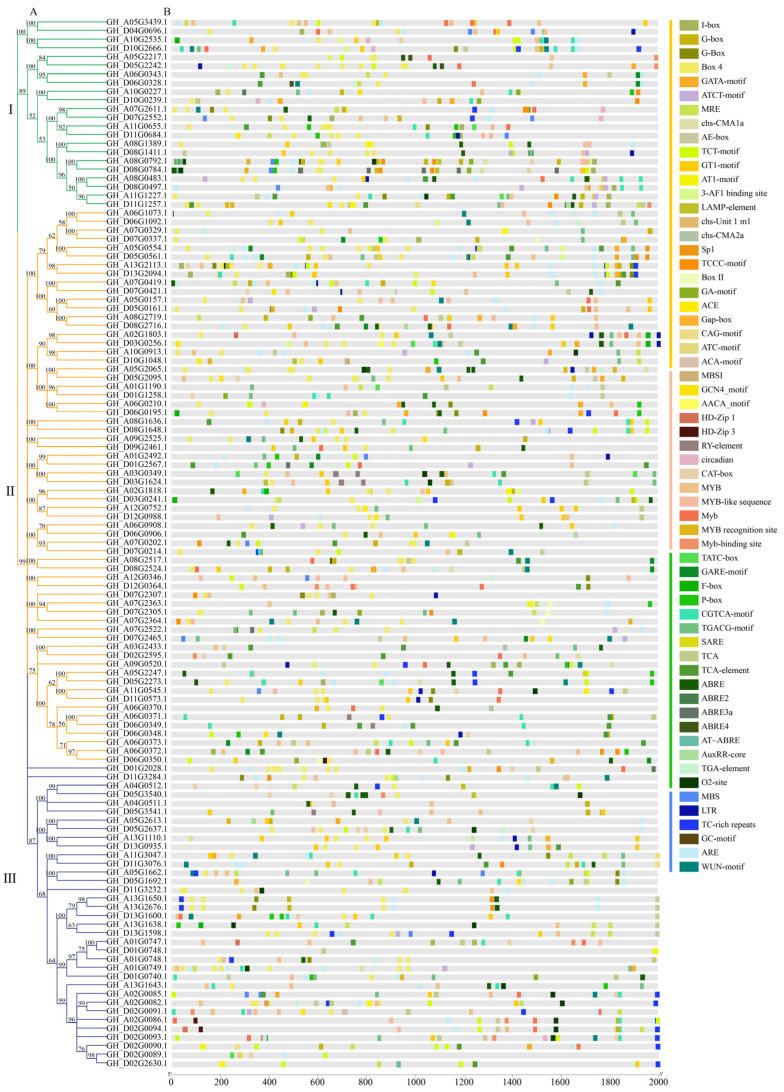
Analysis of cis-elements in promoter regions of *GhFBXLs.* (**A**) Phylogenetic tree of *GhFBXL* genes. (**B**) Analysis of cis-elements in *GhFBXL*s promoters. (I: Grpup I; II:Group II; III: Group III).

The location in the gene promoter region of the cis-acting element can be used as a reference for tissue specificity and stress response to different environments. The cis-acting elements of the *FBXL* gene family mainly include the cis-acting regulatory elements involved in plant growth and development, hormonal response, plant defense, and light-responsive elements. For the sake of delve the possible managerial functions of *GhFBXL* genes under hormone regulation pathways and diverse environmental stresses, the 121 *GhFBXL* genes promoter regions of 2000-bp were submitted to the online database (PlantCARE: http://bioinformatics.psb.ugent.be/webtools/plantcare/html/, accessed on 9 September 2021) for the identification of presumptive plants growth, development, abiotic stress, and phytohormones cis-elements. To cis-elements engaged in plant development and growth, the responded elements of light are the most abundant (39.83%) element in 121 *FBXL* genes promoter regions (Figure 6) and were widely distributed throughout the promoter regions. Other related cis-acting elements involved in plant development and growth include MYB, MYB-like sequence, Myb, MYB recognition site, and Myb-binding site, occupy 17.07%. Another category of cis-acting elements abundant in the promoter section of *GhFBXL* genes was hormone-responsive elements, which the ABA-responsive and MeJA-responsive elements comprised 8.76% and 7.07% of total repeats, respectively, (Table S9). These *GhFBXLs* elements were randomly spread in the promoter regions and were forecasted to participate in plant development and growth, stress, phytohormone, and responses (Figure 6).

### 3.7. Gene Expression Profiles of GhFBXLs

**Figure 7:**
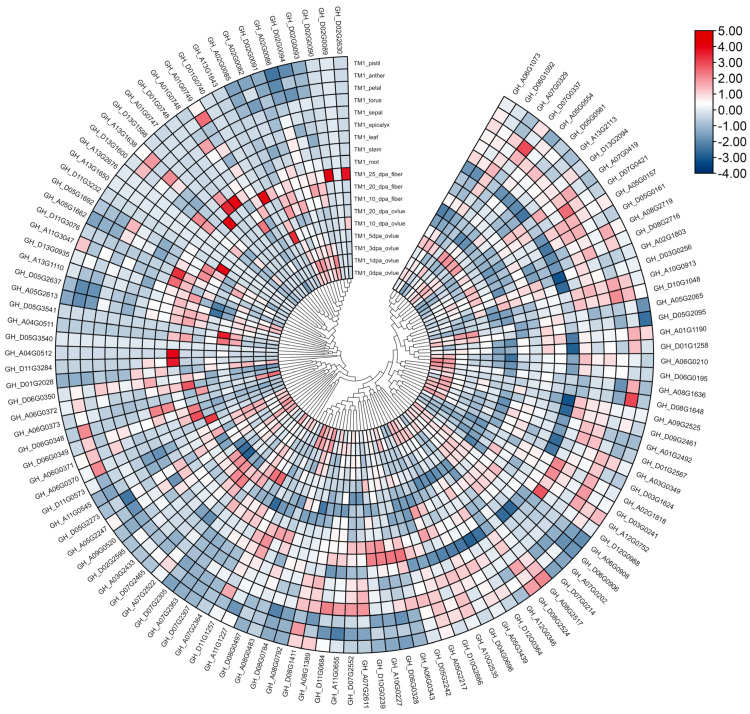
Expression pattern analysis of *GhFBXL* genes. This website’s (http://cotton.zju.edu.cn/, accessed on 15 September 2021) RNA—seq data generated heat maps, and color scale as shown in the figure above on the right side.

To infer the potential biological function of *FBXL*s, we investigated the expression patterns of different *FBXL* genes in *Gh* based on the download from COTTONOMICS (http://cotton.zju.edu.cn/, accessed on 15 September 2021) of the RNA-seq data. Gene expression patterns showed that some *GhFBXL* genes such as *GH_A06G0371*, *GH_A06G0372*, *GH_A07G2522*, *GH_D05G1692*, *GH_D06G0349*, *GH_D07G2465*, *GH_A04G0512, GH_D01G0740*, *GH_D02G2630*, *GH_D02G0090*, *GH_D11G3284*, and *GH_D13G1598* were significantly expressed during the later stage of fiber development (20 DPA: Day Post Anthesis, and 25 DPA fibers). At 10 DPA fibers, remarkable alteration in the expression of two genes (*GH_D11G3232* and *GH_A06G0370*) were observed. The *GH_A13G1643* and *GH_D05G3541* were prominently expressed in ovule at 10 DPA. The two genes (*GH_A07G2364* and *GH_D07G2363*) display a significant expression in 20 DPA ovule. Four genes (*GH_A01G0747*, *GH_D01G0748*, *GH_A11G3047*, and *GH_D11G3076*) were significantly expressed in roots and four genes (*GH_A06G0343*, *GH_D06G0328, GH_A10G0227*, and *GH_D10G0239*) were significantly expressed in stems. Five genes (*GH_A01G0749*, *GH_A08G2719*, *GH_A13G1638*, *GH_D08G1411*, and *GH_D08G1648*) were significantly expressed in anther, while the remaining *GhFBXL*s were lowly expressed or had no expression (Figure 7).

**Figure 8:**
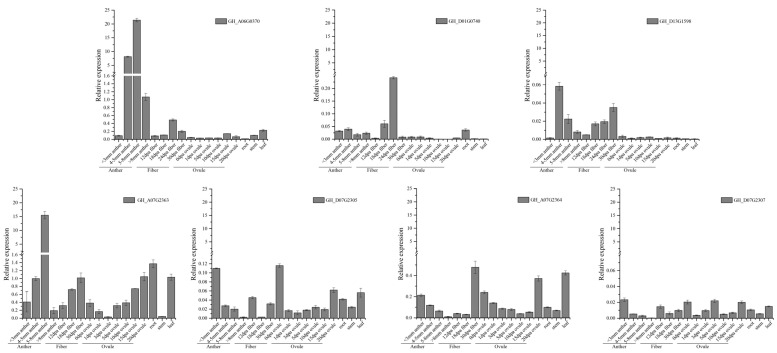
Expression levels of seven *GhFBXLs* in different tissues. *GhActin* was selected as an internal reference for the expression analysis of cotton genes. Error bars represent the standard deviation calculated from three independent experiments.

Using the RNA-seq database, we chose seven *GhFBXL* genes (*GH_A06G0370*, *GH_D01G0740*, *GH_D13G1598*, *GH_A07G2363*, *GH_A07G2364*, *GH_D07G2305*, and *GH_D07G2307*) in different segments to verify the expression profile of *GhFBXL*s by qRT-PCR. The gene expression patterns assessed via qRT-PCR showed a semblable trend to those measured by the RNA-seq data, exhibited in Figure 7 and Figure 8. Amusingly, phylogenetic analysis indicated that *Gorai.001G243000* and *Gorai.001G243100* had the highest homology with *VIT 207s0141g00330* and *AtFBL17*(*AT3G54650*), belonging to subfamily I (Figure 3A). *Gorai.001G243000*, *Gorai.001G243100*, *Ga07G2428*, *Ga07G2429*, *GH_A07G2363*, *GH_A07G2364*, *GH_D07G2305*, *GH_D07G2307*, *GB_A07G2452*, *GB_A07G2453*, *GB_D07G2423,* and *GB_D07G2425* also belong to subfamily I (Figure 3B). Based on the above results, *GH_A07G2363*, *GH_A07G2364*, *GH_D07G2305*, and *GH_D07G2307* may have the same functions as *AtFBL17* (*AT3G54650*), participating in the growth and development of flowering plants, such as twin sperm cell production, double fertilization, cell proliferation, and internal replication [21,22].

### 3.8. Gene Interaction Network of FBXLs

**Figure 9:**
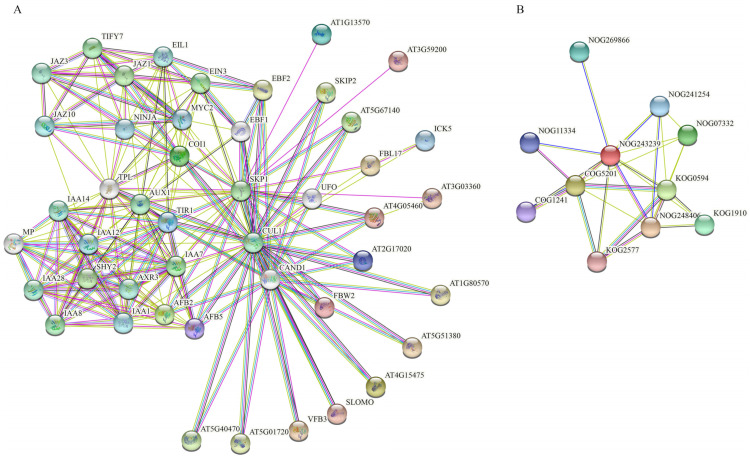
Interaction network of FBXLs. (**A**) Interaction network of GrFBXL and AtFBXL proteins with other proteins. (**B**) Interaction network of FBXL protein families. Nodes’ annotation is shown in Table S11.

For the sake of analysis of the FBXL protein function, we used multiple sequences (Figure 9A) and a protein family (Figure 9B) search to analyze the interaction network by the STRING website (https://string-db.org/, accessed on 19 April 2022), based on Arabidopsis homologous genes.

Using the method of the multiple sequences search, we found many plant hormone-related proteins in the interaction network, such as *AUX1*, *AXR3*, *EIL1*, *EIN3*, *IAA1*, *IAA12*, *IAA14*, *IAA28*, *IAA7*, *IAA8*, and *JAZ1,* among others. As an essential protein for male fertility, *FBL17* is closely related to SKP1 (S-phase kinase-associated protein 1) and UFO (Protein UNUSUAL FLORAL ORGANS); it is also indirectly related to other plant hormones such as auxin (Figure 9A). Using the method of the protein family multiple sequences search, the generative cell mitosis signaling pathway (NOG243239) was discovered in the center (Figure 9B), and other pathways that were pertinent to the phenotypes, such as the regulation of cyclin-dependent protein serine/threonine kinase inhibitor activity (NOG248406), suggesting that the FBXL protein plays a crucial part in the regulation of cell cycle. Transcription factor E2F/dimerization partner (TDP) (KOG2577) was found around the interaction network, a pivotal regulator of cell cycle progression [67]. It is speculated that cotton *FBL17* genes have similar roles in the cell cycle.

## 4. Discussion

The LRR gene family is classified into different subfamilies such as NBS-LRR (nucleotide-binding site leucine-rich repeat), LRR-RPs/LRR-RLPs (leucine-rich repeat receptor/receptor-like proteins), LRR-RKs/RLKs (leucine-rich repeat receptor/receptor-like kinases) and FBXL (F-box/LRR-repeat protein), based on the LRRs (leucine-rich repeats) conserved domain presence at the C-terminus. The LRR domain plays a vital role in defense mechanisms of plants. The main characteristics of LRR genes are the presence of a 20–30 aa structural domain. The F-box protein family is one of the biggest protein families containing members from two in yeast to several hundred in different eukaryotes. The bipartite structure is present in the F-box proteins [14]. Several F-box proteins such as SCF components have been identified in the past few years. F-box proteins, on account of the C-terminus motif, have been classified into three subfamilies i.e., the Fbw or FBXW subfamily contains Kelch, Armadillo and the tetratricopeptide repeats domain at C-terminus, the Fbx or FBXO subfamily had the proline-rich domain, and third Fbl or FBXL subfamily comprises LRR domains at C-terminus of the F-box proteins [10,68].

FBXL is an important F-box subfamily and is reported in many plants such as Arabidopsis (160), rice (61), *M. truncatula* (53), soybean (46), apple (34), chickpea (32), and maize (16) [4,11]. Former research indicated that plant FBXLs’ mediated target proteins degraded in hormonal signals and responded to developmental [12,69]. The FBXL gene family plays a significant role in various aspects of plant growth, such as the hormone-related *TIR1* and *EBF1*, *EBF2* of FBXL gene family members in Arabidopsis [70,71,72], the growth and development-related genes *OE9*, and the *FBL17* control of cell cycle regulation and double fertilization in Arabidopsis [21,22,28]. The information about the role of *FBXLs* in the life cycle of the cotton plant is very limited, but before exploring the biological function of the *FBXLs* in cotton, its comprehensive genome-wide analysis is very important. Here, we present a detailed report of the FBXL gene family in cotton in terms of its phylogeny relationship, motif structure, gene structure, chromosome location, duplication events, and expression analysis. Gene structure analysis of 160 Arabidopsis *FBXL* genes found in previous studies, screening sequences with F-box at the N-terminus and LRR at the C-terminus and identified 127 Arabidopsis *FBXL* genes. The 127 Arabidopsis FBXL protein sequences and 592 *G. hirsutum* F-box protein sequences were used as queries and they identified 121, 119, 57, and 51 *FBXL*s in *Gh*, *Gb*, *Ga*, and *Gr*, respectively (Figure 3). The C-terminus of cotton FBXL protein also had the same pattern of the LRR domain as reported in yeast, bacteria, animals, and plants. LRRs are present in various proteins but their function in plant species is highly conserved. This provides a platform for specific PPI [70], which is very significant for protein function because they recognized ligand or other compositions of the docking pathway [73]. Phylogenetic analysis, motif structure and gene structure analysis indicated that Arabidopsis *TIR1* (AT3G62980) and cotton homologous genes were located in the one branch and had similar gene structure. Arabidopsis *FBL17* (AT3554650) and cotton homologous genes also had semblable gene structure and were located in a branch. We speculate that *TIR1* and *FBL17* in cotton may have the uniform function as Arabidopsis thaliana, which can provide a basis for the study in the development and the fusion of male and female gametes [21,22,71].

When comparing the *FBXL* genes with other studied plant species, we found that *FBXL* gene numbers in *G. hirsutum* (121) and *G. barbadense* (119) are the second and third largest after *A. thaliana* (127). The number of *FBXL* genes is species-specific but extraneous with the genome scale. The large variation in the *FBXLs* among different plant species is due to bountiful increases or losses of *FBXL*s. Gene loss and repetition events also happened in the cotton genome, which results in the underrepresentation of the *FBXL*s in cotton. The *G. hirsutum* is the best example of domesticated plant polyploidy and genome-scale duplication because it is the hybrid product of *G. raimondii* and *G. arboretum*, but slight gene loss also occurs during the cotton polyploidization. Previous studies suggested that the WGD event is the main source of increase in the gene members of the family in *G. hirsutum*. Comparison with the 121 *FBXL*s in the *G. hirsutum* genome, the 119 *FBXL*s in the *G. barbadense* genome, 57 *FBXL*s in *G. arboretum*, and 51 in *G. raimondii* genome were also observed (Figure 3), which suggested that slight change in FBXL family members also occur as done for other families in the cotton genome after the evolution process from diploid to tetraploid. Replication modes besides WGD contributed to the increase of the FBXL members in cotton. The increase in the FBXL family members in cotton indicated that the FBXL family is essential to enhance the different characters and adaptation of upland cotton in response to various environmental factors.

Different PPI domains are present at the C-termini of the F-box, which is known to interact with different substrates [4,11]. Domain analysis of the cotton FBXL genes revealed the presence of three types of LRR domains such as a, b, and c at their C-terminus, allowing their classification into three groups (Figure 1). In four cotton species, 46.26% of the predicted genes were found in LRR domain, 30.46% genes contained type b, and 23.28% genes had type c domain (Table S6). Similarly, *FBXL* genes with FBD domains at C-terminal were also observed in other species like chickpea (39), rice (9), and *M. truncatula* (139) [4,74,75]. FBD is the plant-specific domain of F-box proteins. Although, the precise function of the FBD domain is unknown but thought to be associated with a biochemical process in the nucleus [74]. The cotyledon vein patterning may be associated with the FBXL protein-containing, plant-specific FBD domain by setting the biochemical base for the interplay between the protein with other unidentified cell components. Therefore, it is suggested that FBXL may be correlated with cotyledon vein patterning [34].

The intron—exon arrangement of FBXL family members confirmed the presence of 10.48% intron-free genes in the gene family, which is also a prominent character of the FBXL gene family as also observed in the Arabidopsis, rice, *M. truncatula*, soybean, apple, chickpea, maize, respectively [4,11,74,75,76,77,78]. In addition, we observed that the intron—exon structure of most of the subfamily members are the same (Figure 2), which suggests the close structural relationship among the *FBXL* genes within the subfamily. The phylogenetic tree among the cotton FBXL proteins was built to research the evolutionary relation. It divides the FBXL proteins into three clades. The arrangement of the FBXL proteins through phylogenetic analysis showed that *FBXL*s with semblable C-terminal domains co-evolved as observed in other plant species. The identical domain arrangement was observed in the members of each clade which suggested that they interact with a similar substrate for function. A similar phylogenetic arrangement was observed in soybean [46], chickpea [74], Arabidopsis [11], and rice [4] suggesting the gene family has a common evolutionary origin in dicots and monocots.

The gene duplication event is a momentous source of increasing the number of family members and variety of functions during the evolution process across chromosomal segmental duplication or tandem duplication [79]. Erstwhile studies showed that the replication event is a significant method of gene family amplification [80]. Moreover, *FBXLs’* expansion is also the consequence of the replication event. The duplication analysis of the FBXL protein family revealed that 222 of 229 (96.94%) *FBXL* genes undergo a duplication event. The segmental/WGD duplication was observed in 152 genes (68.47%), while 41 (18.47%) genes arose through tandem duplication (Tables S4 and S5), suggesting that segmental/WGD duplications influence surpassed tandem duplication in the FBXL gene family expansion in cotton. A similar observation was also found in soybean [46], chickpea [74], Arabidopsis [11], and rice [4], indicating that plant genomes follow the same mechanism for the duplication event of *FBXL* genes. The strong tendency for negative selection in the F-box domain and positive selection in the C-terminal domain of the genes was reported during the evolutionary process, which leads to the F-box domain having sequence conservation and the C-terminal domain having sequence variation [81,82]. This was also the cause for the remarkable variation in F-box protein length, as reported in different plant species [80]. This may also be due to the amino acid gain or loss within the F-box protein for resilient evolution to recognize different substrates.

In the upstream promoter region of the *GhFBXL* genes many cis-elements were found that related to phytohormones signals and environmental stress, most of which were Abscisic acid, Salicylic acid, and auxin; these are essential for the anaerobic induction element (Figure 6 and Table S9). Cyclin-dependent protein serine/threonine kinase inhibitor activity (NOG248406) and numerous phytohormones related proteins (such as *AUX1*, *IAA1*, *IAA12*, etc.) and plant reproductive development-related protein (*FBL17* and *UFO*), have been found in the gene interaction network, which provides strong support for the role of *FBXL* genes in growth and development [21,22,25].

## 5. Conclusions

In this study, we preliminarily analyzed the gene sequence, exon—intron structure, conserved motifs, phylogenetic relationships, gene collinearity, Ka/Ks selective pressure, and gene expression profiles of cotton *FBXL* genes. It laid the foundation for the study of *FBXL* genes in cotton growth and development, such as floral organ development, male and female gametophytes, and male sterility. Further research on *TIR1, FBL17*, and other *FBXL* genes in cotton may reveal the auxin regulation mechanism and cultivate male sterile lines in cotton.

## Data Availability

The data presented in this study are available in the Supplementary Materials.

## Supplementary Materials

The following are available online at https://www.mdpi.com/article/10.3390/genes13122194/s1. Table S1: The characterization of four cotton species *FBXL* genes in this study. Table S2: The gene structure of three cotton species *FBXL* genes in this study. Table S3: Phylogenetic analysis of *FBXL* gene numbers among different species. Table S4: The number of duplication types of *FBXL* gene among different cotton species. Table S5: Duplication types of the *FBXL* Gene in different Cotton species. Table S6: The domain of four cotton species *FBXL* genes in this study. Table S7: The number of *FBXL* genes on chromosomes among different cotton species. Table S8: Ka/Ks values of duplicated *FBXL* gene pairs from three cotton species. Table S9: Analysis of *GhFBXL*s cis-elements. Table S10: The primers used for qRT-PCR in this study. Table S11: Notes on nodes of FBXL proteins interaction network. Figure S1: The conserved motifs, Exon–intron structures of *G. arboretum FBXLs*. Figure S2: The conserved motifs, Exon–intron structures of *G. raimondii FBXLs*. Figure S3: The location of *FBXL* genes on Ga, Gr, Gh, and Gb.
