# Supplementary figures and images for: Identification, Classification and Characterization Analysis of FBXL Gene in Cotton
Source: Genes (Basel). 2022 Nov 23;13(12):2194. doi: 10.3390/genes13122194 (PMC9777894; doi:10.3390/genes13122194)

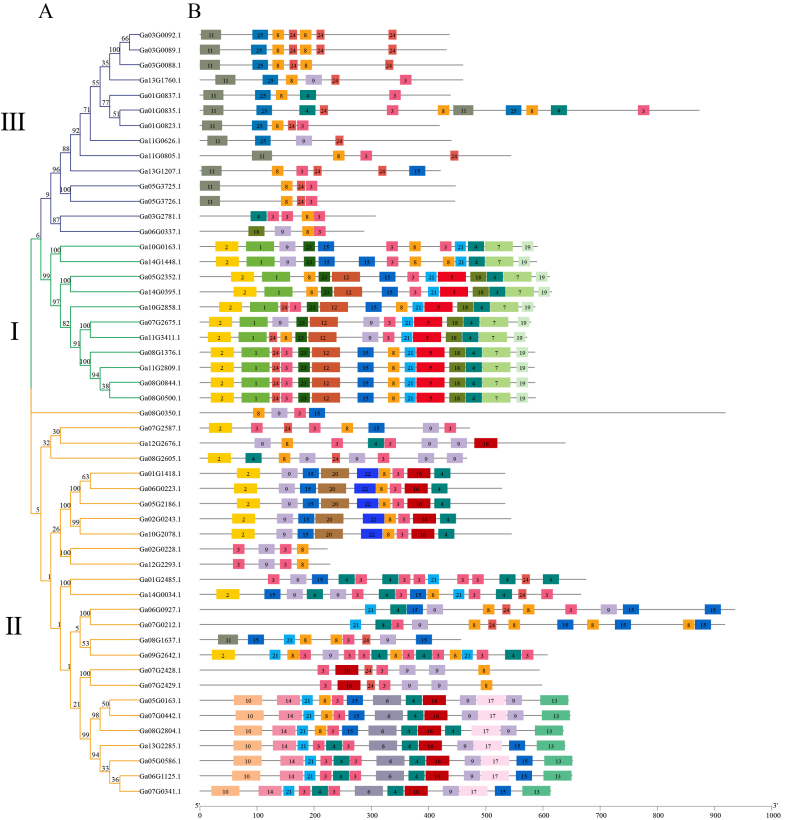

Supplement: Supplementary file 1 [file genes-13-02194-s001.zip › genes-1932548-supplementary/Supplementary Figure 1.The conserved motifs, Exon¿Cintron structures of G. arboretum FBXLs..pdf]

## Ga

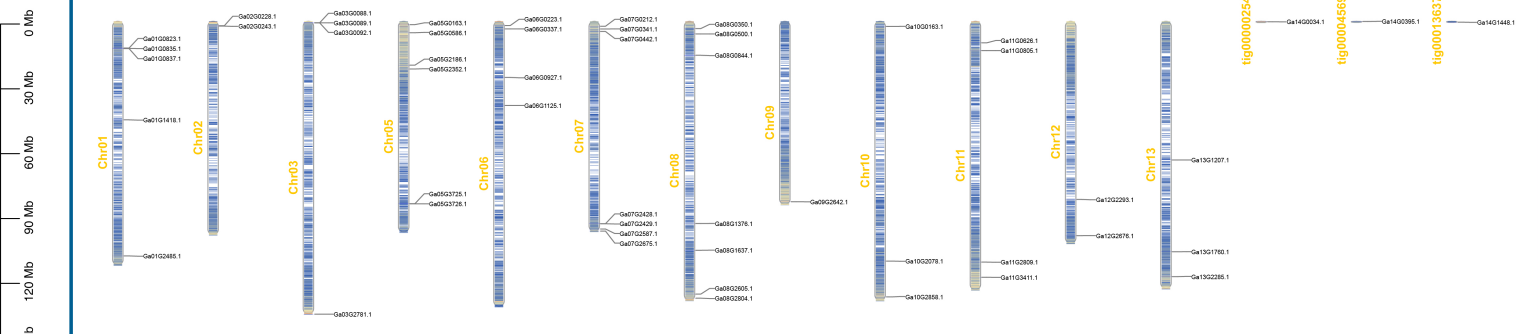

## Gr

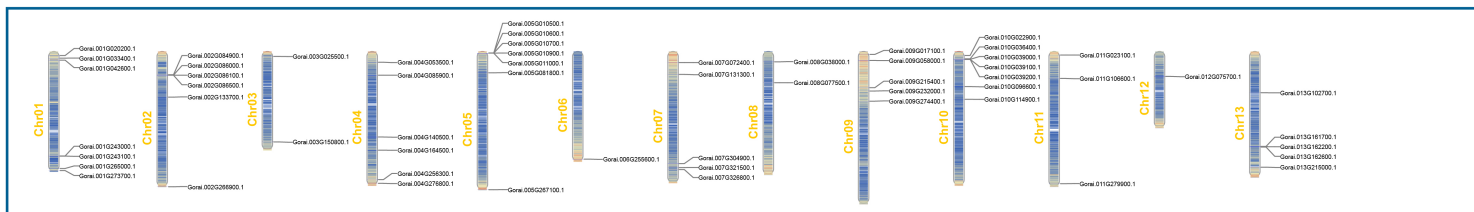

## Gh

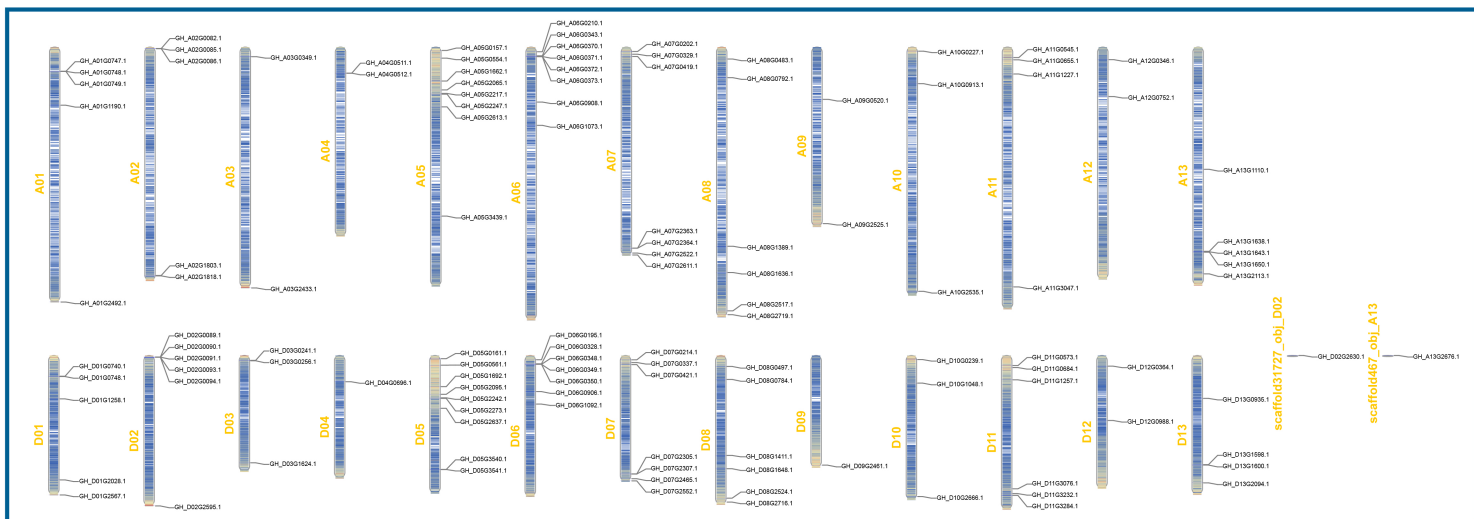

## Gb

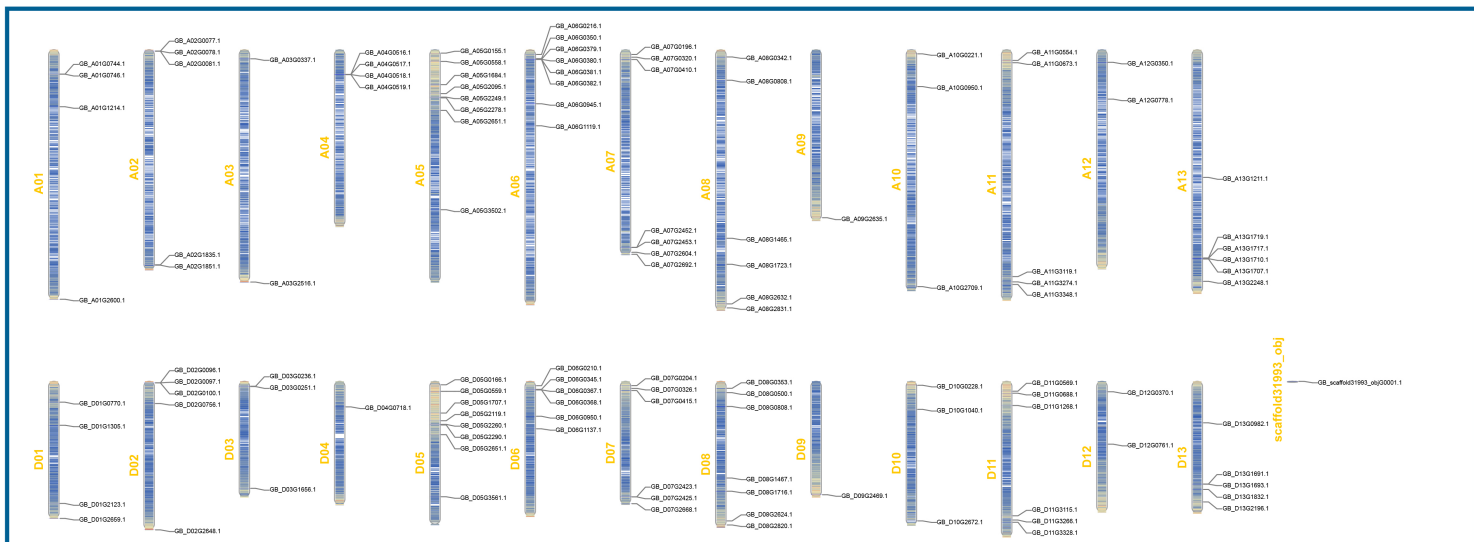

Supplement: Supplementary file 1 [file genes-13-02194-s001.zip › genes-1932548-supplementary/Supplementary Figure 3.The location of FBXL genes on Gaú1⁄4 Grú1⁄4 Gh, and Gb..pdf]
