# Supplementary material for: Identification, Classification and Characterization Analysis of FBXL Gene in Cotton
Source: Genes (Basel). 2022 Nov 23;13(12):2194. doi: 10.3390/genes13122194 (PMC9777894; doi:10.3390/genes13122194)
Supplement: Supplementary file 1 [file Supplementary Figure 2.The conserved motifs, Exon¿Cintron structures of G. raimondii FBXLs..pdf › genes-1932548-supplementary/Supplementary Figure 2.The conserved motifs, Exon¿Cintron structures of G. raimondii FBXLs..pdf]

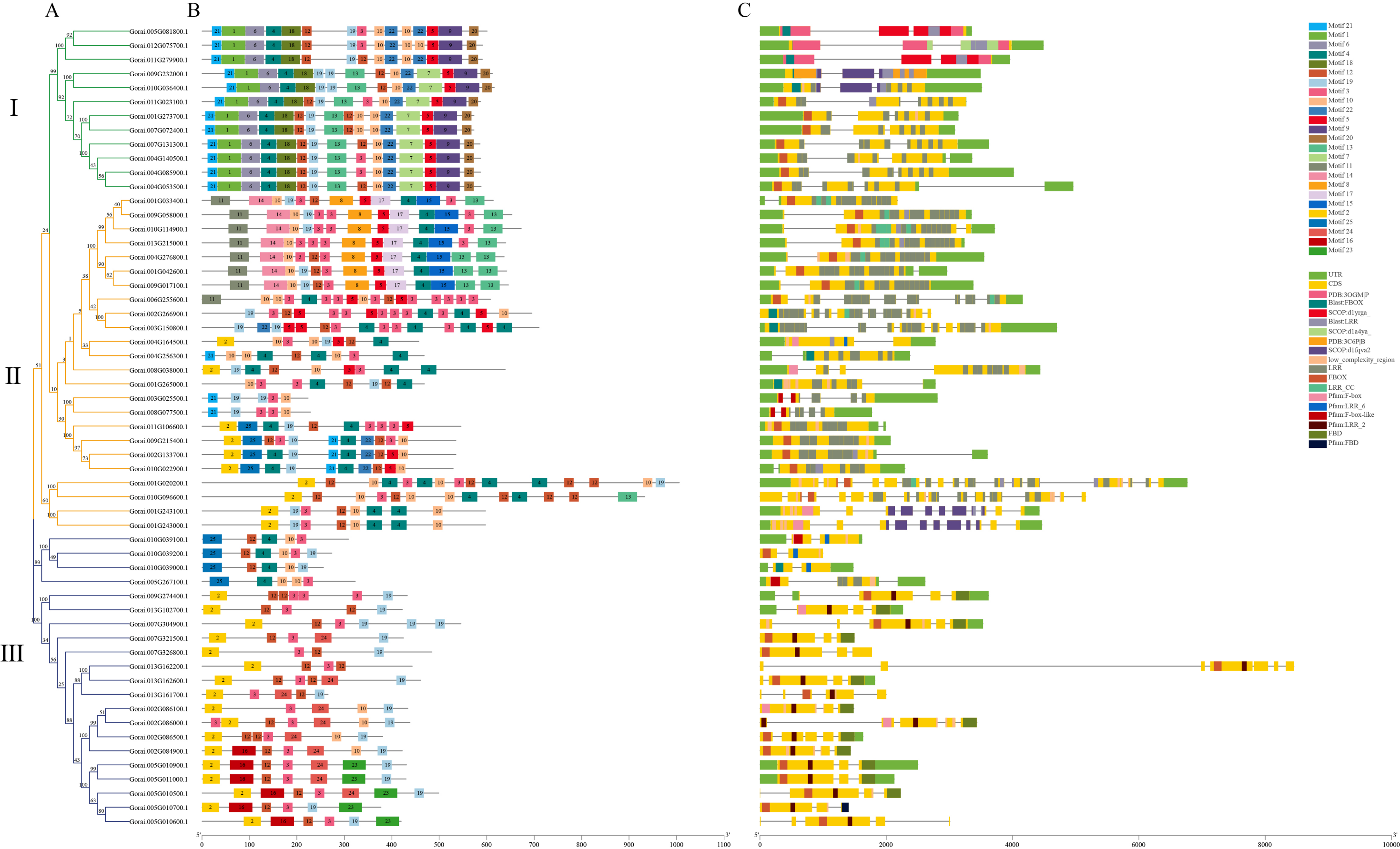

A
B
C
I
II
III
Gorai.005G081800.1
Gorai.012G075700.1
Gorai.011G279900.1
Gorai.009G232000.1
Gorai.010G036400.1
Gorai.011G023100.1
Gorai.001G273700.1
Gorai.007G072400.1
Gorai.007G131300.1
Gorai.004G140500.1
Gorai.004G085900.1
Gorai.004G053500.1
Gorai.001G033400.1
Gorai.009G058000.1
Gorai.010G114900.1
Gorai.013G215000.1
Gorai.004G276800.1
Gorai.001G042600.1
Gorai.009G017100.1
Gorai.006G255600.1
Gorai.002G266900.1
Gorai.003G150800.1
Gorai.004G164500.1
Gorai.004G256300.1
Gorai.008G038000.1
Gorai.001G265000.1
Gorai.003G025500.1
Gorai.008G077500.1
Gorai.011G106600.1
Gorai.009G215400.1
Gorai.002G133700.1
Gorai.010G022900.1
Gorai.001G020200.1
Gorai.010G096600.1
Gorai.001G243100.1
Gorai.001G243000.1
Gorai.010G039100.1
Gorai.010G039200.1
Gorai.010G039000.1
Gorai.005G267100.1
Gorai.009G274400.1
Gorai.013G102700.1
Gorai.007G304900.1
Gorai.007G321500.1
Gorai.007G326800.1
Gorai.013G162200.1
Gorai.013G162600.1
Gorai.013G161700.1
Gorai.002G086100.1
Gorai.002G086000.1
Gorai.002G086500.1
Gorai.002G084900.1
Gorai.005G010900.1
Gorai.005G011000.1
Gorai.005G010500.1
Gorai.005G010700.1
Gorai.005G010600.1
5'
3'
0 100 200 300 400 500 600 700 800 900 1000 1100
0 2000 4000 6000 8000 10000
Motif 21
Motif 1
Motif 6
Motif 4
Motif 18
Motif 12
Motif 19
Motif 3
Motif 10
Motif 22
Motif 5
Motif 9
Motif 20
Motif 13
Motif 7
Motif 11
Motif 14
Motif 8
Motif 17
Motif 15
Motif 2
Motif 25
Motif 24
Motif 16
Motif 23
UTR
CDS
PDB:3OGM|P
Blast:FBOX
SCOP:d1yrga_
Blast:LRR
SCOP:d1a4ya_
PDB:3C6P|B
SCOP:d1fqva2
low_complexity_region
LRR
FBOX
LRR_CC
Pfam:F-box
Pfam:LRR_6
Pfam:F-box-like
Pfam:LRR_2
FBD
Pfam:FBD
